# Epidemiological Study of the Incidence of Cancers Eligible for Proton or Carbon Ions Therapy: Methodology and Results of Recruitment Estimation

**DOI:** 10.1155/2013/107646

**Published:** 2013-06-20

**Authors:** Stéphanie Patin, Pascal Pommier, Hu Yi, Marie Hélène Baron, Jacques Balosso

**Affiliations:** ^1^GCS ETOILE, 60 Avenue Rockefeller, 69008 Lyon, France; ^2^Centre Régional de Lutte Contre le Cancer Léon Bérard, 28 Rue Laennec, 69008 Lyon, France; ^3^CHU Jean Minjoz, 25030 Besançon Cedex, France; ^4^Université Joseph Fourier, Grenoble1, BP 53-38041, Grenoble Cedex 9, France

## Abstract

*Context*. Hadrontherapy is an innovative form of radiotherapy using beams of protons or carbon ions able to destroy some radio-resistant tumours. Because these tumours are highly specific amongst all cancerous tumours, it is impossible to determine the incidence of these diseases from surveillance registries. *Goal*. To assess, within the Rhône-Alpes region, the incidence of cancers being hadrontherapy indications. *Method*. Prospective, multicentre continuous data collection during 1 year, by practitioners participating to multidisciplinary tumor board. Tumours are inoperable, radio resistant, at primary stage of development, or locally recurrent, with low metastatic potential. *Results*. Study involved 27 healthcare centres, 52 groups of specialist practitioners. The estimated incidence of cancers eligible for hadrontherapy in the Rhône-Alpes region in 2010, that is, for 34 locations in all, is of 8.5/100 000 inhabitants. Appraisal of the low potential of metastatic progression is impeded, because these are rare diseases, whose outcome is unfamiliar to investigators. *Conclusion*. Future epidemiological studies will need to focus on prognosis and on the metastatic progression rate of these diseases. Indeed, there are few information available on this subject in the literature that could be used to improve preventive measures, medical care, and the surveillance of these rare cancers.

## 1. Scientific Context

### 1.1. Hadrontherapy

Hadrontherapy is an innovative form of radiotherapy, based on high-technology equipment using proton or carbon ions beams to destroy tumours [1, 2]. This treatment method enables significantly higher ballistic precision to be achieved, compared to photons (X-rays) with, as expected therapeutic benefit, an improvement of quality of life and chances of recovery [3]. Carbon ions are also specifically characterised by superior biological efficacy (relative biological effectiveness from 1.5 to 3), overcoming the radiation resistance of certain cancers to photons and even protons. Indeed, carbon ion beams when compared to X-rays represent a distinct advantage for the treatment of highly radiation-resistant tumours [4].

### 1.2. Hadrontherapy Epidemiological Studies

An initial study assessing recruitment potential for proton therapy was conducted in 1998 in Italy, showing an incidence of 10 825 cases/year [5]. One year later, a second study was carried out in the context of the MedAustron project for the construction of a carbon ions therapy centre in Austria. Considering patients living in Austria and neighbouring countries, the patient recruitment potential (proton and carbon) was estimated with an incidence of 13 145 cases/year [6]. In France, in 2002, a third study was conducted in the context of the ETOILE medical project. This study revealed an annual incidence of approximately 5320 cases/year for carbon ions [7]. Concomitantly, a fourth study was conducted, again in the context of the MedAustron project, estimating the incidence of cases eligible for hadrontherapy in Austria at 2044 cases/year [8]. Finally, again in 2002, in the context of the project for the construction of the CNAO centre in Italy, an epidemiological study was conducted, estimating the incidence of cases eligible for carbon therapy at 3694 cases/year and proton therapy at 1885 cases/year. [9]. Since 2004, no other epidemiological studies have been published for hadrontherapy.

In 2011, however, the Japanese team from the National Institute for Radiological Sciences (NIRS), pioneer in carbon therapy, published the results of its 1994 to 2011 clinical activity. Patients were included in clinical trials and the number of patients receiving carbon therapy was 6157 [10]. This value is the incidence of treated cases, over 17 years without any notion of recruitment potential and with a rather slow ramp up of the activity. 

### 1.3. Therapeutic Indications for Hadrontherapy

**Figure 1:**
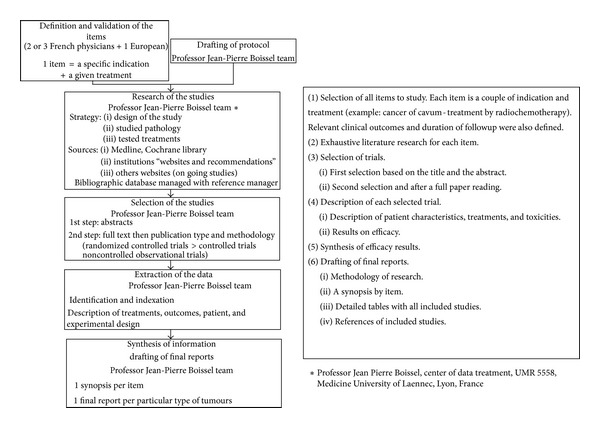
Literature review method.

A list of priority indications that can be treated by hadrontherapy (Table 1) was drawn up by the GCS ETOILE medical group, in collaboration with national and international experts, based on published data ranked using an “evidence-based medicine” type analysis (Figure 1). This literature review was based on provided or expected medical service criteria (survival and quality of life). According to the classification used by the European Survey Group of Rare Diseases [11], the eligible tumours that can be treated by hadrontherapy conform to the criteria of rare diseases based on the following thresholds of epidemiological indicators: prevalence ≤ 50 cases/100 000 persons within a given population and incidence ≤ 6 cases/100 000 persons/year. Indeed, for all tumor types identified during the previous hadrontherapy epidemiological studies [5–8], the calculated and cumulative incidences are all below these thresholds (Table 2). Amongst the hadrontherapy indications, 25 are tumours listed in the ORPHANET international database of rare diseases [12] (cf. ORPHANET number in Table 1). 

### 1.4. Problem

The exact incidence of each disease eligible for hadrontherapy is difficult to determine from the available resources. Indeed, the published data [5–13] pertain to series of patients for which the aim was not to assess the level of demand for treatment. These were rather series of patients who had received the therapy, but whose recruitment had not been accounted for in tumour registries. Considering the scarcity and specific nature of the tumours concerned by hadrontherapy, it is difficult to extrapolate and assess the treatment demand from these results. Hadrontherapy indications are defined according to anatomical location of the tumours, clinical stage, pathology, therapeutic alternatives (e.g., surgical contraindications), and patient characteristics (general condition and comorbidities). French cancer registries record data required to estimate cancer incidence but fail to provide sufficient detail of the tumour stage or of therapeutic data. Moreover, the registries cover only approximately 15% of the French population and mainly for the most frequent cancers. Therefore, these registries do not allow the incidence of these tumours to be estimated.

### 1.5. Challenges and Prospects

This regional study will provide us with more detailed figures concerning the potential number of new cases eligible for hadrontherapy, allowing us to better adapt the future care offer delivered by the national hadrontherapy centre. This survey should also provide us with information on certain medical indicators to assist in the design of future hadrontherapy clinical trials (detailed incidence data, patient population characteristics, tumour stages, recruitment pools, etc.). Finally, this epidemiological field study is also eventually intended to be used to organise a network of highly specialised cares.

### 1.6. Hypothesis

According to the first epidemiological study carried out in France [6], in association with (i) the results of the literature review that defined the list of hadrontherapy indications and (ii) the indications chosen for proton therapy in France, the number of cases in the Rhône-Alpes region eligible for hadrontherapy is estimated at 200 per year (the Rhône-Alpes region is home to 10% of the French population).

## 2. Goals

The main goal is to asses, within the Rhône-Alpes region, the incidence of cancers with hadrontherapy indication.

The secondary goals are (1) to characterise the affected population, (2) to describe the characteristics of the observed cancers, and (3) to describe the characteristics of the treatments implemented when hadrontherapy is unavailable.

## 3. Method

*Study Outline.* This is a prospective, multicentre incidence study, lasting 24 months, conducted with the RCP (*Réunion de Concertation Pluridisciplinaire*—multidisciplinary tumor board) groups of the Rhône-Alpes region in France, that offer a therapeutic strategy for cancers for which hadrontherapy is an alternative.

*Centre Recruitment and Inclusion Method.* This study required the involvement of a part-time consultant epidemiology engineer, who totalled one woman-year of work to provide (1) phone canvassing to identify and involve regional cancer research players; (2) the protocol construction and testing phase, along with the data collection tools; (3) meetings with 3C (cancer coordination centres) coordinators and RCPs; (4) investigator centres inclusion meetings. An extensive number of travels within the region were required for these tasks.

*Variables Collected.* These included year of birth; gender; patient's department of residence; World Health Organization (WHO) performance status, patient's therapeutic status: initial stage or recurrence; initial staging (UICC TNM classification: International Union against Cancer); treatments received before recurrence; time to recurrence; staging at time of current tumour management; ICD-10 (International Classification of Diseases); ICDO (International Classification of Diseases for Oncology) histological type; site of surgery and margin quality; postsurgical histopathological stage (pTNM stage); prior radiotherapy; proposed treatment.

*“Patient” Inclusion Criteria.* The criteria included adults and children, no age limit, and patients presenting with hadrontherapy indications listed in Table 1, whose medical files have been discussed by experts during an RCP. Subjects must be in compatible general and psychological condition. 

*Definition of “Patient” Inclusion Criteria. Compatible general and psychological condition* refers to patients not suffering from life-threatening comorbidities (no acute or chronic diseases whose short-term lethal risk is dominant relative to the cancer), capable of adhering to a disease monitoring protocol and of understanding and accepting a complex treatment requiring close cooperation and staying for several days away from home. 

*“Tumour” Inclusion Criteria.* These include unresectable tumour, belonging to a known radiation-resistant pathological group, mainly in a locoregional development stage, recurrent or local relapse, with low metastatic potential or M+ dissemination (presence of one or more remote metastases at the time of diagnosis) presenting a low threat (slow development and/or metastases that can be readily treated). These inclusion criteria are the results of a consensus synthesis of a systematic literature review performed during 8 years, pathology by pathology with the participation of about one hundred of European medical, surgical, and radiation oncologists, managed by physicians trained in hadrontherapy (mainly protontherapy). The review was carried out by an academic laboratory specialized in clinical trial setup and analysis and was made according to the “evidence-based medicine” principle. 

*Definition of “Tumour” Inclusion Criteria*

- *Radiation resistance:* tumour which estimated local dose required for local control that is higher that the maximum acceptable dose equivalent (MPDE) for surrounding organs necessarily irradiated under applicable technical conditions, assessed by a radiation oncologist (guide des Procédures de Radiothérapie Externe 2007 (guidelines for external radiotherapy procedures); joint effort by the French society for oncology radiotherapy (société Française de radiothérapie oncologique—SFRO), and the French society for medical physics (société française de physique médicale—SFPM), conducted in collaboration with the representatives of the French association of electroradiology paramedical workers (association française du personnel paramédical d'electroradiologie—AFPPE)).
- *Unresectable:* tumour which local or locoregional expansion stage renders excision either is technically impossible (opinion of an experienced surgical team) or surgically unacceptable due to the irreparable damage that would be necessary (cancer: Principles & Practice of Oncology 2005; DeVita, V. General articles on treatment strategies in cancer).
- *Locoregional:* it refers to the tumour expansion state corresponding to the anatomical diffusion region of the primary tumour by contiguous expansion (T of TNM) and/or by lymphatic diffusion, maintaining a rank of N in TNM staging, in other words, any tumour at stage M0 (latest edition of the tumour TNM classification published by the (UICC) International Union against Cancer or the (AJC) American joint committee for cancer staging).
- *Low metastatic potential:* M0 situations whose medium-term (5-year) metastatic risk is considered to be sufficiently low to justify curative locoregional treatment involving significant means. This threshold is difficult to determine; a level greater than 50% seems unreasonable. As an example, mucosal melanomas are just at this threshold.
- *Nonthreatening M+ dissemination or controlled by medical treatments:* this means the presence of one or more remote metastases at the time of diagnosis, presenting no immediate threat as displaying slow development and/or accessible to effective treatment (typical case of some low-grade sarcomas lung metastases). These situations are indicative of locoregional disease treatment using complex and expensive techniques such as hadrontherapy.
- *Local relapse or local recurrence (these two terms are synonymous):* it means the redevelopment, at the same site as initially, of a previously effectively treated tumour (dictionary of medical terms, Garnier-Delamare).

*Primary Endpoint.* it is the quantitative estimate of the incidence of indications eligible for hadrontherapy.

*Secondary Endpoints.* They are the description of the tumours and population characteristics, along with the treatments proposed and implemented by the RCPs. 

*Types of Investigation Centres and Participant.* Those were healthcare establishments (private and public) in the Rhône-Alpes region hosting RCP groups and having radiotherapy departments.

*Types of RCP Groups Participating in Screening.* they consisted of groups of oncology specialists with expertise in the following diseases: musculoskeletal sarcomas and tumours, Head and Neck (H and N), gastroenterology, paediatrics, dermatology, endocrine and central nervous system tumours.

*Case Screening Modalities.* Case screening is performed continuously by the physicians and clinical research technician in light of the list of indications (Table 1), during each RCP.

*Data Collection Modalities.* Data are collected in a decentralised prospective manner on paper questionnaires. 

*Study Monitoring.* Fifteen percent of files were checked to ascertain data accuracy. 

*Study Quality.* The questionnaire, along with the organisation of the investigation, was assessed beforehand by a sample population of investigators (3C coordinators and CRAs) via a semiguided telephone interview. For the questionnaire, the evaluation focused on general understanding, the form and length of the questionnaire, time required for completion, amount of data to collect, and the relevance of the selected criteria. The evaluation of the investigation method pertained to the choice of individuals involved in data collection organisation, the data recording method, the organisation for data recording, and the identification source for clinical cases. 

*Data Quality.* Variables are encoded in a uniform and standard manner in an investigator's guide book, which contains detailed information concerning the definitions of each indicator to collect. Dual computer data input is performed, along with checks for missing or improbable data (encoding errors, date inconsistencies).

*Statistical Methods.* No sampling is performed as this study is intended to be exhaustive. The incidence of cancers is expressed as the number of cases reported from the demographic figures of the Rhône-Alpes region over the studied period and expressed per 100,000 inhabitants. Estimations of endpoint descriptive characteristics are expressed as frequencies, means, and percentages. Analyses are performed using Microsoft Excel, version 2008. 

## 4. Results

 The study involves 13 clinical research technicians, 50 RCP coordinating physicians and 12 cancer coordination centres (see Table 3). The study mobilises 27 Rhône-Alpes region healthcare centres, of which 8 university hospitals, 1 regional cancer centre, 1 public-private cancer institute, 12 general hospitals, the Lyon paediatric oncology hospital institute, and 4 private clinics. The study test phase validated the tools and organisation of this investigation. This led to a sense of ownership and motivation on the part of the centres. As a whole, the study covered the region's 11 hospital areas and involved 52 groups, out of some sixty listed, of cancer specialists with expertise in the diseases that were to identify.

During the study, 53 cases eligible for hadrontherapy were identified. Mean patient age was 43 years [2–89], and men were more numerous (OR = 1.52). The highest number of cases (39.6%) was in the 2–18 years age group. The number of patients with recurrence was slightly lower (45.3%) than the number of patients receiving initial care (54.7%). Carcinomas, sarcomas (Table 4), and paediatric tumours (Table 5) were the most frequently identified diseases, and their locations are varied. During the RCP, radiotherapy, chemotherapy, and surgery were prescribed, respectively, in 58%, 57%, and 57% of cases. No brachytherapy was prescribed.

For the group of 45.3% relapsing patients, previous treatments were chemotherapy, surgery, or conventional radiotherapy in 62.5%, 58.3%, and 58.3% of cases, respectively.

**Figure 2:**
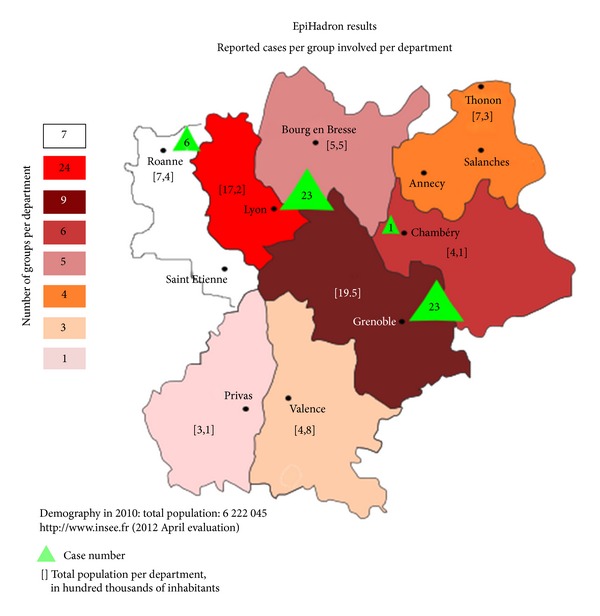
Results, EpiHadron study: number of cases identified relative to the number of groups involved in each department.

The number of cases identified during the study was approximately four times lower than the potential estimate. (Figure 2) Data collection was nonhomogeneous. The Isère department identified 43.4% of cases (i.e., 2.5 cases/RCP), and the Rhône department identified 56.6% (i.e., 1.2 cases/RCP). Isère identified twice as many cases as Rhône, even though it only has nine expert groups, that is, 2.6 times less than in Rhône, which has 24. The geographic areas not covered by the study correspond to the location of 15 private healthcare centres and to the medical activity of six general and four specialist RCP groups. The first six groups had no recruitment potential according to the statement of their coordinators. The four other groups had a recruitment potential.

This preliminary data collection, conducted over a one-year period, reported 53 cases of hadrontherapy indications, compared to the estimated cap of 200 cases. The Rhône-Alpes region was home to 6,222,045 inhabitants in 2010 [14]. The estimated incidence of cancers eligible for hadrontherapy in the Rhône-Alpes region in 2010, regardless of disease, is of 8.5/100,000 inhabitants. 

## 5. Discussion

### 5.1. About the Method

The diseases to identify in this study are little known, their distribution has not been studied, and we are unaware of any epidemic outbreaks. Moreover, considering that the cancers to identify are rare, it is likely that the number of cases to identify per investigator centre will be very low (estimated incidence of at the most 1 to 5 cases/year). Finally, the primary goal of the RCP groups is to provide an immediate therapeutic solution. Case identification for an observational study is thus unusual in the context of an RCP. Because of this, sustaining team vigilance and motivation for case identification is difficult to obtain and requires regular refreshing throughout the study.

The study test phase validated the tools and organisation chosen for this investigation. This step also served to circulate hadrontherapy information and to facilitate adhesion, ownership, and mobilisation around the project. The individuals involved in study organisation were in charge of systematically directing each cancer patient to the elective experts. Patients suffering from diseases eligible for hadrontherapy were frequently in relapse condition and rather at the end of their therapeutic options. This is due to the fact that these diseases have usually a slow and long evolution; therefore, this study had more chance to catch them somewhere in an advanced stage than at the beginning. It can also be considered that the lack of satisfactory treatment means as hadrontherapy makes these populations have more chance to show more advanced disease than it will be expected to be seen in the future. Their management requires discussion by a medical team of cancer specialists. The identification and decision to include a patient in the study were made by the RCP groups including these experts. Under these conditions, we have confidence in the case orientation and identification system.

The “tumour with low metastatic development potential” selection criterion is one of the criteria used to identify patients eligible for hadrontherapy. In our current state of knowledge, however, this indicator gives clinicians the greatest difficulties. 

The results of this study were obtained mainly from public hospitals. This can be explained in part by the fact that it is easier to create multidisciplinary expert groups in public hospitals and to set up clinical research. Due to their structure, these establishments benefit from human resources with more varied medical specialities than in private radiotherapy practices. In all likelihood, cases eligible for hadrontherapy could have been treated by these private centres during the study and ignored. 

One should be very cautious when comparing the results of this study with those of published incidence studies. The methodologies employed in these previous studies are heterogeneous, and/or their flow is insufficiently documented. Moreover, their goal was to estimate a recruitment potential based on the extrapolation of the results. The methodologies used were not intended to provide information concerning an exhaustive incidence, as proposed by our study. Finally, the published studies were conducted in attractive institutions, specialising in cancer care and selected for this reason; this may bias the assessment of prevalence. Under these conditions, it is easy to confuse incidence and prevalence. It is thus conceivable that the estimation of incidence in the literature is overestimated. These elements could, in part, explain the observed difference between the preliminary results obtained in our study and the estimate calculated from the epidemiological results published in the literature. Only the publication of results obtained from other exhaustive epidemiological studies conducted in the same areas could support this hypothesis. 

### 5.2. About the Results

It is unlikely that the lack of case recording associated with the medical activity of the four groups not involved in this data collection could alone explain the observed difference between the calculated incidence estimates and the preliminary results obtained. For several clinicians involved in the study, the prognosis and risk of metastatic development were difficult to estimate considering the lack of knowledge for these rare diseases. Moreover, the novelty of hadrontherapy, which can potentially modify usual treatment strategies, could have led to the omission of this indication in some cases, in particular conditions unusually treated by radiotherapy as hepatic and biliary tumours. These difficulties, along with the more sustained vigilance effort made by some groups relative to others, could account for the nonhomogeneous data collection and the observed difference between predictions and obtained results, despite the high motivation of the investigators. Furthermore, the reorganisations (case of two major RCP clusters), changes of persons involved and the low frequency of cases to report, lead to a constant erosion of vigilance, requiring continuous action by the organiser.

This work aimed to structure a network of care pathway. However, some important institutions with their own recruitment targets did not wish to participate, which reduces the scope of our results.

About the detailed conditions of the recruited cases, there are a large proportion of recurrent diseases as explained above, and some metastatic diseases have also been included according to defined characteristics: (i) the number of metastasis was limited (1 to 3 metastasis); (ii) their very slow growing speed was not immediately threatening; (iii) the possibility of effective treatment of these “pauci-metastatic” conditions, essentially by ablative procedures. Actually, the principle to treat, with curative intend, patients in such situation is more and more accepted by expert oncologists.

The estimated incidence of cancers, irrespective of type and location, eligible for treatment by hadrontherapy, for the study, that is, 8.5/100,000 individuals, is mildly higher than the reference incidence threshold of ≤6 cases/100,000 individuals, as defined in the ORPHANET international database of “rare diseases” [12]. This figure can be obviously explained by the fact that the incidence was calculated by adding the five major cancer categories and 34 different locations. If one was to calculate the incidence for each of the disease types, their respective incidence would be significantly below the reference threshold of ≤6 cases/100,000. Indeed, for our study, the minimum number of cases listed per disease is of 1 case and the maximum is of 7 cases. Table 4.

Considering all of the difficulties and limitations of this preliminary phase of the study, the yield of approximately 25% notification, compared to the expected maximum, appears satisfactory. In the perspective of a (necessary) ramp up of a future hadrontherapy centre, there is thus a sufficient population to initiate operation and to progressively establish recruitment for this centre, without immediately generating an insufficient offer effect.

## 6. Conclusion

Future epidemiological studies will benefit from focusing on the characterisation of the metastatic development of diseases eligible for hadrontherapy. All cancers have their own development pattern, and, in this respect, the literature provides few elements concerning diseases eligible for hadrontherapy. This information should serve both to improve diagnosis procedures, medical care, and the surveillance of these rare diseases. 

This study of hadrontherapy healthcare decision networks in the Rhône-Alpes region, able to identify areas not properly covered by RCPs, should enable us to analyse the potential effect of this lack on the outcome of the patients of these areas. 

Incidence studies will be insufficient to determine the actual impact of hadrontherapy activity. These studies provide condensed elements about the medical activity associated with new patients only. They shed no light on posttreatment medical activity: follow-up visits, medical imaging, and other care beyond the irradiated activity per se.

One of the most valuable outcomes of this study has been to make the existence of some particularly rare diseases known and to provide information concerning an innovative treatment: hadrontherapy, whose existence will doubtless have a favourable effect on the very knowledge of these diseases. An additional benefit of this approach has been to open the way for a new treatment system in a region of France that is home to approximately 10% of the country's population. At last, these elective indications will have to be validated by health authorities to register them as part of the good professional practices of oncology and give an equal chance to each patient to receive hadrontherapy through the well-established decision process of the RCPs.

## Figures and Tables

**Table 1:** Ranked list of hadrontherapy indications.

Type	Location	Clinical situations	ORPHANET number	European prevalence/100,000/year/2011*
Head and neck (H and N)	Salivary glands	Inoperable/refusal of surgery/R2 resections/local relapses/all histology		
	Facial sinuses	Inoperable/refusal of surgery/R2 resections/local relapses/all histology		
	Cystic adenoid carcinoma, base of skull expansion	Inoperable/refusal of surgery/R2 resections/local relapses		
	Malignant mucosal melanoma	Any location without immediately threatening metastases Tumour if possible nonoperated or R2 or nonirradiated local relapse	ORPHA168999	46.8
	Squamous cell carcinoma	Relapses or second unresectable location in irradiated area and M0	ORPHA67037	<40

Skull brain spinal column	High-grade glioma (grade III or glioblastomas)	Relapse after initial radiotherapy treatment ± chemotherapy and progressing under chemotherapy. Initial treatment possibly after surgery and other EG inoperable in R2	ORPHA360	1
	Benign meningioma	Base of skull, convexity	ORPHA2495	
	Malignant meningioma	Base of skull, convexity	ORPHA2495	
	Pituitary adenoma	Base of skull, to large for surgery or stereotactic rT	ORPHA99408	
	Ependymoma	Brain, posterior fossa, spinal column	ORPHA301	
	VIII neurinoma	Base of skull, to large for surgery or stereotactic rT	ORPHA637	
	Local and meta medulloblastoma	CNS	ORPHA616	
	Chordoma and sarcoma	Base of the skull, spinal column and sacrum: All clinical forms	ORPHA178	

Digestive	Single hepatocarcinoma	*∅* > 4 to 5 cm, unresectable, M0, untreatable by conventional methods or photon therapy, with no life-threatening comorbidities	ORPHA88673 ORPHA210159 ORPHA33402	1
	Single nodular cholangiocarcinoma	unresectable, M0, not previously irradiated, and nonprogressive under chemotherapy for 4 to 6 months	ORPHA70567	
	Pancreatic adenocarcinoma	unresectable, M0, not previously irradiated, and nonprogressive under chemotherapy for 4 to 6 months	ORPHA217074 ORPHA1333	11.9
	Endocrine tumours of the pancreas (M0)	M0, progressive after multiple treatments: isotopic and/or chemotherapy and somatostatin	ORPHA97253	
	Pelvic relapse of rectal cancer	M0, previously irradiated or not	

Sarcoma and chordoma	Nonretroperitoneal soft tissue	Low grade and M0, all histologies, all sites. Unresectable, surgery refused, or “definitive R2”: R2 without possible revision, R2 after revision surgery, or local R2 relapse. Nonthreatening M+ situation with debilitating T or rT		
	Retroperitoneal sarcoma	After local relapse and revision surgery: “R0” or R1 and M0 (for unresectable and R2 Ts, see above). Or R1 M0 situation		
	Head, neck, and limb soft tissues	“Definitive R1”: R1 resection without acceptable possibility of surgery	ORPHA97338	
	Osteo- and chondrosarcoma (excluding axial skeleton)	Nonoperated tumours or R2, M0 resections. M+ accepted for osteosarcomas only. Discussion according to grade	ORPHA223727	
	Chordoma and sarcoma	Base of the skull, spinal column, and sacrum: all clinical forms	ORPHA178	

Paediatric tumours	Pelvic Ewing's sarcoma	inoperable, voluminous (more than 100 or 200 mL according to age)	ORPHA319	0.1
	Aggressive chordoma in young children	<3-4 years	ORPHA178	
	Osteosarcoma	pelvic, unresectable	ORPHA668	
	Cranial and parameningeal sarcoma	Skull and base of skull		
	Primitive ectodermal tumour	M0 or inoperable	ORPHA251870	
	Medulloblastoma	Posterior fossa + medullary irradiation	ORPHA616	
	Craniopharyngioma		ORPHA54595	
	Ependymoma		ORPHA301	
	Optic pathway glioma		ORPHA2086	
	Neuroblastoma	M0 and inoperable	ORPHA635	11.3
	Retinoblastoma		ORPHA790	5.4

Other	Miscellaneous, highly functional sites, meeting the study inclusion criteria	Criteria to declare in appendix 4 (study questionnaire)		

Legend: R0: complete tumour resection without residues; R1: tumour resection with microscopic residues; R2: tumour resection with macroscopic residues; EG: conditions; CNS: central nervous system; *∅*: circumference; M0: medium-term metastatic risk (within 5 years) and considered to be sufficiently low to justify locoregional treatment; M+: several metastases at time of initial diagnosis, leading to discussion of locoregional treatment; T: tumour; rT: radiotherapy.

*Estimated incidences according to the ORPHANET 2011 survey [12].

**Table 2:** Results, EpiHadron study: summary of incidence rates calculated during hadrontherapy epidemiological studies conducted between 1998 and 2004.

Country	Gross incidence/year*	Incidence/ /100,000 inhabitants/year*	Total population during the year of the study (ù)	Study year	Related healthcare centre
Italy	10,825	19	56 908 265	1998	CNAO
Austria	4873	61	7 982 461	1999	Med-AUSTRON
Switzerland	263	4	7 123 537	1999	Med-AUSTRON
Slovenia (a)	626	31	2 000 092	1999	Med-AUSTRON
Hungary	153	1	10 253 400	1999	Med-AUSTRON
Italy	313	1	56 913 634	1999	Med-AUSTRON
Czech Republic (b)	5060	50	10 190 000	1999	Med-AUSTRON
Slovakia (y)	1734	32	5 477 038	1999	Med-AUSTRON
France	5320	9	61 424 036	2002	ETOILE
Austria	2044	25	8 063 640	2002	Med-AUSTRON
Italy	5579	10	57 888 245	2004	CNAO

*Estimate expressed in study population, irrespective of disease location.

(y, a, b) Estimate of 2011, no data available before this date.

(ù) Demographic sources.

http://www.statistiques-mondiales.com.

http://appsso.eurostat.ec.europa.eu/nui/setupModifyTableLayout.do.

http://www.diplomatie.gouv.fr.

**Table 3:** Results, EpiHadron study: organisation, patient, and tumour characteristics.

Categories	Population sizes (*n*)	%
Organisations		
Investigating establishments	27	—
RCP groups: multidisciplinary consultation meeting	52	—
Cancer coordination centres	12	—
RCP coordinating physicians	50	—
Clinical research associate	13	—
Patient characteristics		
Patient age (mean)	43 years [2–89]	—
Man	32	60.4
Woman	21	39.6
Initial care	29	54.7
Recurrent cancer	24	45.3
Tumour characteristics		
Mean tumour size (largest circumference)	69 mm [19–250]	—
Metastases present	11	20.8
Lymph nodes present	10	18.9
Identified Cases		
In total (*N*)	53	—
By age group		
[2–18] years	21	39.6
[18–45] years	0	0
[45–65] years	18	34
[65–69] years	0	0
[69–89] years	14	26.4
By RCP type		
Head and neck H and N	4	7.5
Digestive	12	22.6
Sarcoma and soft tissue	2	3.8
Central nervous system CNS	2	3.8
Paediatrics	21	39.6
Dermatology	4	7.5
General practice	6	11.3
Oncology	1	1.9
Other	1	1.9

**Table 4:** Results, EpiHadron study: list of cases identified by anatomical type.

Type	Category	Location	Population sizes (*n*)	%	C/P
Carcinomas	Adenocarcinoma	of the endometrium	1	1.9	C
		of the pancreas	1	1.9	C
		of the rectum	7	13.2	C
		caecum with invasion of the psoas	1	1.9	C
	Cystic glandular	of the left parotid gland	1	1.9	C
		of the arytenoid cartilage	1	1.9	C
	Epidermoid	of the scalp	1	1.9	P
		oesophagus	2	3.8	P
		retromolar or intermaxillary commissure	1	1.9	P
		oropharynx	1	1.9	P
	Other	basal cell	1	1.9	C
		of the rectum	1	2.3	C
		neuroendocrine pancreatic	1	1.9	C
Total carcinomas			**20**	**38.2**	
Sarcomas	Osteosarcoma	of the humerus, upper end	1	1.9	C
		thigh and leg bone or cartilage	1	1.9	C
		osteogenic of the calvarium	1	1.9	C
	Leiomyosarcoma	of the mucosal maxillary sinus	1	1.9	C
	Chondrosarcoma	of the larynx	1	1.9	P/C
	Skin	posterior basicervical	1	1.9	C
	Liposarcoma		1	1.9	C
	Ewing's		5	9.4	P/C
	Rhabdoïd	clival	3	5.7	C
	Grade II myxofibrosarcoma	antero-external part of the left leg	1	1.9	C
Total sarcomas			**16**	**30.2**	
Skull Brain and Spinal column	Neuroblastoma	of the base of the skull	1	1.9	P
	Glioma	of the brain stem nuclei	1	1.9	P/C
		of the optic nerve	1	1.9	P
		hypothalamic-chiasmatic	1	1.9	P
	Glioblastoma		3	5.7	C
	Grade IV medulloblastoma	of the posterior cerebral fossa	2	3.8	P
	Craniopharyngioma		1	1.9	C
	Malignant melanoma	of the left maxillary sinus	1	1.9	C
	Xanthoastrocytoma	of the temporal lobe	1	1.9	P/C
Total brain			**12**	**22.6**	
Other	Merkel cell tumour	of the left buttock	1	1.9	C
	Chordoma	sacrum	2	3.8	C
	Adrenal tumours		2	3.8	C
Total other			**5**	**9.4**	

Totals (*N*)			53	100	

Legendary: C: carbon, P: proton.

**Table 5:** Results, EpiHadron: list of cases identified by anatomical type for paediatric tumours.

Paediatric tumours	Category	Location	Population sizes (*n*)	%
Neurological tumours	Neuroblastoma	The base of the skull	1	4.8
	Neuroblastoma	Right adrenal gland	1	4.8
	Neuroblastoma	The adrenal glands	1	4.8
	Grade IV medulloblastoma	The posterior cerebral fossa	2	9.5
	Glioma	The brain stem lymph nodes	1	4.8
	Glioma	The optic nerve	1	4.8
	Glioma	Hypothalamic-chiasmatic	1	4.8
	Craniopharyngioma		1	4.8

Sarcomas	Osteosarcoma	Thigh and leg bone and cartilage	1	4.8
	Osteosarcoma	Upper end of the humerus	1	4.8
	Osteogenic Osteosarcoma	The calvarium	1	4.8
	Ewing's sarcoma		5	23.8
	Rhabdoid sarcoma		1	4.8
	Xanthoastrocytoma	Temporal neurological	1	4.8
	Rhabdoid tumour	The clival region	2	9.5

Total paediatric tumours			21	100
